# GhWRKY40 Interacts with an Asparaginase GhAP_D6_ Involved in Fiber Development in Upland Cotton (*Gossypium hirsutum* L.)

**DOI:** 10.3390/genes15080979

**Published:** 2024-07-24

**Authors:** Sujun Zhang, Xiao Cai, Jingyan Wei, Haitao Wang, Cunjing Liu, Xinghe Li, Liyuan Tang, Xiaodong Zhou, Jianhong Zhang

**Affiliations:** 1 Institute of Cotton, Hebei Academy of Agricultural and Forestry Sciences, Shijiazhuang 050051, China; sujun1987_good@126.com (S.Z.); caixiaoziji@163.com (X.C.); wanghaitao6488@126.com (H.W.); cunjingliu@sohu.com (C.L.); xingheli111@126.com (X.L.); liyuaner05@163.com (L.T.); zhouxd87@hotmail.com (X.Z.); 2 Key Laboratory of Biology and Genetic Improvement of Cotton in Huanghuaihai Semiarid Area, Ministry of Agriculture and Rural Affairs, Shijiazhuang 050051, China; 3 National Key Laboratory of Cotton Bio-Breeding and Integrated Utilization, Institute of Cotton Research, Chinese Academy of Agricultural Sciences, Anyang 455000, China; weijingyan@caas.cn

**Keywords:** fiber quality, GhWRKY40, aspartyl protease, yeast two-hybrid assay, split-luciferase complementation assay, *Gossypium hirsutum*

## Abstract

Fiber quality improvement is a primary goal in cotton breeding. Identification of fiber quality-related genes and understanding the underlying molecular mechanisms are essential prerequisites. Previously, studies determined that silencing the gene *GhWRKY40* resulted in longer cotton fibers; however, both the underlying mechanisms and whether this transcription factor is additionally involved in the regulation of cotton fiber strength/fineness are unknown. In the current study, we verified that GhWRKY40 influences the fiber strength, fiber fineness, and fiber surface structure by using virus-induced gene silencing (VIGS). Potential proteins that may interact with the nucleus-localized GhWRKY40 were screened in a yeast two-hybrid (Y2H) nuclear-system cDNA library constructed from fibers at 0, 10, and 25 days post-anthesis (DPA) in two near-isogenic lines differing in fiber length and strength. An aspartyl protease/asparaginase-related protein, GhAP_D6_, was identified and confirmed by Y2H and split-luciferase complementation assays. The expression of *GhAP_D6_* was approximately 30-fold higher in the *GhWRKY40*-VIGS lines at 10 DPA and aspartyl protease activity was significantly upregulated in the *GhWRKY40*-VIGS lines at 10–20 DPA. This study suggested that GhWRKY40 may interact with GhAP_D6_ to regulate fiber development in cotton. The results provide a theoretical reference for the selection and breeding of high-quality cotton fibers assisted by molecular technology.

## 1. Introduction

Breeders aim to improve fiber quality while maintaining high yield potential and stability. Cotton fibers are single-celled protrusions from the epidermal cells of ovules. Fiber development can be classified into four overlapping periods, namely, initiation, elongation, secondary cell wall (SCW) biogenesis, and dehydration maturity [1]. It is usually considered that the fiber length and fiber numbers are largely determined in the initiation and elongation periods, which are approximately 0–15 days post-anthesis (DPA). In comparison, the SCW biogenesis period is associated with fiber strength and fineness at 20–40 DPA [1,2]. Fiber quality is a genetically complex quantitative trait, controlled by multiple genes and affected by environmental factors [3]. Researchers have mapped 701 quantitative trait loci (QTLs) for fiber length and 919 QTLs for fiber strength, using intraspecific and interspecific populations based on data from the CottonGen website (https://www.cottongen.org/ accessed on 6 June 2024) [4]. Given the negative genetic association between fiber quality and yield [5], it is critical to investigate fiber quality-related genes and understand the intricate genetic relationships to enhance fiber quality [6]. During fiber development, a large number of factors, such as transcription factors, plant hormones, and membrane proteins, can affect the final fiber quality [7], and the regulatory effects of different factors and different time periods can affect each other, thus forming a complex regulatory network. Many potential fiber-related candidate genes have been identified with whole-genome sequencing [8,9,10,11,12]. Among them, some important transcription factors involved in the development of cotton fibers have been validated, such as *MYB* transcription factors *MYB_A12*, *GhMML3*, and *GhMML7* [13,14] and *bHLH* transcription factors *GhbHLH18*, *GhTCP2*, and *GhTCP9b* [15,16]. Among the genes known to affect cotton fiber quality [17], the WRKY transcription factors have not been studied sufficiently.

WRKY DNA-binding proteins are among the largest transcription factor families in plants and play important roles in plant development and stress response [18,19]. Several studies have examined the role of WRKY transcription factors in cotton fiber development [20]. For instance, WRKY gene subgroup III is primarily expressed in ovules or fibers at −3 to 3 DPA, and heterologous expression of *GhWRKY53* in *Arabidopsis thaliana* significantly increases epidermal hair density [21]. *GhWRKY16* plays a role in cotton fiber initiation and elongation because the number of fibrous protrusions on ovules of *GhWRKY16*-silenced plants is significantly reduced and the fibers are shorter compared with the control [20]. *GbWRKY40* is speculated to be involved in the development of cotton fiber secondary walls in *Gossypium barbadense* because it is highly expressed in fibers at 25 DPA [22]. Recently, we observed that *GhWRKY40* was likely to participate in cotton fiber elongation [23]. However, both the underlying mechanisms and whether this transcription factor is additionally involved in the regulation of cotton fiber strength are unknown.

Protein–protein interaction is a powerful approach to studying molecular mechanisms of gene actions. Yeast two-hybrid (Y2H) systems are a common strategy to detect protein–protein interactions in vivo [24], and several tissue-specific cDNA libraries have been constructed as collections of capture proteins that interact with target proteins in cotton [25]. In the present study, the functions of *GhWRKY40* in cotton fiber strength and fiber fineness were verified using virus-induced gene silencing (VIGS), and potential downstream proteins that may interact with GhWRKY40 were screened in a Y2H nuclear-system cDNA library from fibers at 0, 10, and 25 DPA in two near-isogenic lines differing in fiber length and strength, from which *GhWRKY40* was originally identified. As a result, an aspartyl protease/asparaginase-related protein, GhAP_D6_, was identified and confirmed by Y2H and split-luciferase complementation assays. Aspartyl protease activity was significantly upregulated in the *GhWRKY40*-VIGS lines at 10–20 DPA. The results suggested that GhWRKY40 may interact with aspartyl protease to regulate fiber development in cotton. Ultimately, these molecular resources will assist in molecular breeding to develop high-quality cotton fibers.

## 2. Methods and Materials

### 2.1. Plant Materials

We selected two near-isogenic lines, A1 and A29, derived from the same *G. hirsutum* L. hybrid cotton variety Ji1518 and differing in fiber length and strength, as materials in this study (Table 1). The breeding of the two lines is detailed in a previous publication [23].

### 2.2. VIGS Assay

The plasmids pCLCrVA:*GhWRKY40* (*Ghi_D05G02861*), pCLCrVA:*00* (negative control), and pCLCrVA:*GhCLA1* (silencing effect indicator) were transformed into the *Agrobacterium tumefaciens* strain GV3101, which was then infiltrated into two fully expanded 10-day-old cotyledons of A29 (with shorter and weaker fibers). When the plants entered the flowering stage, the stem tip was infiltrated every 3 weeks to maintain the silencing effect. The VIGS assay procedure is described in detail in a previous publication [23]. When the new leaves of the pCLCrVA:*GhCLA1* plants became white, 2–3 VIGS bolls per plant (at 10 DPA) were confirmed using quantitative real-time PCR (qRT–PCR). The central bolls that bloomed within 3 days from 10–15 plants were collected and combined as one replicate, with three replicates collected in total. We assessed three fiber quality-related indicators, namely, *F*_max_ (the force required to break a fiber, or cN), tenacity (the fiber-breaking tenacity, or cN/tex), and linear density (Lin.Den, the fiber fineness, or dtex), of each replicate with the FAVIMAT (Textechno, Mönchengladbach, Germany) instrument at the Inspection and Test Center of Cotton Quality affiliated with the Ministry of Agriculture and Rural Affairs of China (Anyang, Henan Province, China). A total of one hundred fibers were individually tested per biological repeat. The significance of differences between the pCLCrVA:*GhWRKY40* and pCLCrVA:*00* lines (*p* < 0.01) was assessed with SPSS software 18.00. The quality-related data were visualized using imageGP (http://www.ehbio.com/ImageGP/index.php/Home/Index/Boxplot.html accessed on 6 June 2024).

### 2.3. Scanning and Transmission Electron Microscopic Analysis

Samples of mature fibers from the *GhWRKY40-VIGS* and negative-control (pCLCrVA:*00*) lines were used for scanning electron microscopic (SEM) analysis and transmission electron microscopic (TEM) analysis.

For SEM analysis, samples were attached directly to the conductive adhesive and sprayed with gold for 45 s using a Quorum SC7620 sputter coater at 10 mA and imaged using a MIRA LMS (TESCAN, Kohoutovice, Czech Republic) scanning electron microscope at 3.0 kV.

For TEM analysis, transverse sections of the fiber samples were fixed in 2.5% glutaraldehyde and incubated at 4 °C for 12–24 h and then in 1% OsO_4_ for 2 h. The samples were dehydrated through an ethanol gradient series and embedded in Spurr’s medium prior to ultrathin sectioning. Sections (70–90 nm thick) were cut with an ultramicrotome (EM UC7, Leica, Wetzlar, Germany) and transferred onto copper grids. The sections were air dried, stained, and viewed with a Hitachi H-7650 transmission electron microscope at 80 kV (Shiyanjia; https://www.shiyanjia.com accessed on 6 June 2024). A range of 5 to 10 non-serial sections per genotype from fibers were examined per line [26].

### 2.4. Subcellular Localization

The whole-genome resequencing data for A1 and A29 (https://www.ncbi.nlm.nih.gov/bioproject/PRJNA1019498/ accessed on 6 June 2024) revealed no variation in the coding sequence (CDS) of *GhWRKY40*. The *GhWRKY40* CDS was synthesized by Sanggong (Shanghai, China) and then was cloned into the N-terminal fusion green fluorescent protein (GFP) vector pR101-GFP. This construct was introduced into the *A. tumefaciens* strain GV3101 and then was infiltrated into the leaves of *Nicotiana benthamiana* plants. The subcellular localization of the GFP signal was detected within 48 h of infiltration using an Olympus FV1200 confocal microscope. The marker PBIN-RFP was used as a nuclear marker.

### 2.5. Construction of Cotton Fiber Nuclear-System Y2H Library

The total RNA from the cotton fibers of A1 and A29 at 0, 10, and 25 DPA was extracted separately, and then equal amounts of RNA from each sample were mixed to obtain the total fiber RNA for Y2H library construction. The CloneMiner™ II cDNA Library Construction Kit (A11180, Invitrogen, Shanghai, China) was used to construct the cDNA library with the Gateway^®^ Technology (Shanghai, China) (www.lifetechnologies.com/support accessed on 6 June 2024). The primary library employed the pDONR222 vector and the secondary library used the pGADT7-DEST vector. Specific steps comprising synthesis of the first strand, synthesis of the second strand, ligation of the attB1 adapter, size fractionation of the cDNA by column chromatography, the BP recombination reaction, the transformation of competent cells, and the plating assay were described previously [26]. The titer for each plate was calculated with the following formula: titer (cfu/mL) = colonies on plate × dilution factor/volume plated (mL). The titer for each plate was used to calculate the average titer for the entire cDNA library. The total number of colony-forming units (CFU) was calculated with the following formula: total CFU (cfu) = average titer (cfu/mL) × total volume of cDNA library (mL). In total, twenty-four clones were randomly selected for determination of the recombination rate and insertion target length. The qualified secondary library plasmid was transformed into the yeast strain Y187 (630457, Clontech, Beijing, China).

### 2.6. Screening GhWRKY40-Interacting Proteins Using the Cotton Fiber Y2H Library

*GhWRKY40* was cloned into the bait vector pGBKT7 at the *Eco*RI/*Bam*HI restriction sites. The pGBKT7-GhWRKY40 bait plasmid and pGADT7-T were co-transformed into yeast cells to perform self-activation and toxicity tests on the dropout medium (SD/−Trp; DDO: SD−Trp/−Leu; TDO: SD/−Trp/−Leu/−His; QDO: SD/−Trp/−Leu/−His/−Ade) (BD Biosciences Clontech, Palo Alto, CA, USA). The plasmids pGBKT7-Lam control vector + pGADT7-T control vector were used as a negative control, whereas the plasmids pGBKT7-53 control vector + pGADT7-T control vector were used as a positive control. The pGBKT7-GhWRKY40 bait plasmid was used to screen the Y2H library of cotton fiber cDNAs on TDO plates. Positive clones were selected and sequenced by Sangon Biotech (Shanghai, China). The cDNA sequence similarities were analyzed by blasting the data against the NCBI public databases. Expressed sequence tags (ESTs) for each gene were collected and classified.

### 2.7. Y2H and Split-Luciferase Complementation Assays

To validate interactions between GhWRKY40 and its targets, full-length cDNAs of the candidate-interacting proteins were amplified and cloned into the pGBKT7 or pGADT7 vectors for one-to-one verification with the Y2H assay. The primers used to amplify the genes are listed in Table S1. Co-transformed yeast clones were serially diluted (1:10) and spotted on DDO and QDO media for growth assessment [27].

For the split-luciferase complementation assay, the full-length cDNA of GhWRKY40 and each of the interacting proteins were cloned separately into the N-terminus and C-terminus of the pCAMBIA1300 fusion vector. The plasmids were co-expressed in *N. benthamiana* leaf cells after *Agrobacterium*-mediated infiltration. A pair of interacting proteins, GmZF351+GmZF392, was used as a positive control [28]. A plant in vivo imaging system (Berthold LB 985) was used to observe the fluorescence signal after incubation for approximately 48 h.

### 2.8. Verification of Candidate Genes by qRT–PCR

The qRT–PCR analysis was performed using the ABI Prism 7500 system (Applied Biosystems, Foster City, CA, USA). *GhHistone3* (GenBank accession: AF024716) was used as the internal reference gene and the 2^−ΔΔ*C*t^ method was used to calculate the relative expression level. The primers used are listed in Table S1. Three biological and technical replicates were analyzed. The significance of any differences was assessed at a threshold of *p* < 0.05.

### 2.9. Aspartyl Protease, Peroxidase, and Indoleacetic Acid Oxidase Activities

After confirming the significant downregulation of *GhWRKY40*, fibers were collected separately at 10, 15, and 20 DPA from the *GhWRKY40-VIGS* lines and negative control lines to determine the activities of aspartyl protease, peroxidase (POD), and indoleacetic acid oxidase (IAAO). The activity of aspartyl protease was determined using the Suzhou Grace Biotechnology Company Kit (spectrophotometric method) in accordance with the instruction manual (aspartyl protease activity: JM110533P2; POD: JM01185P1; IAAO: JM110305P1). Three replicates per sample were analyzed, and the significance of any differences was assessed at a threshold of *p* < 0.05.

## 3. Results and Discussion

### 3.1. GhWRKY40 Was Involved in Fiber Strength and Fiber Fineness in the VIGS Assay

**Figure 1:**
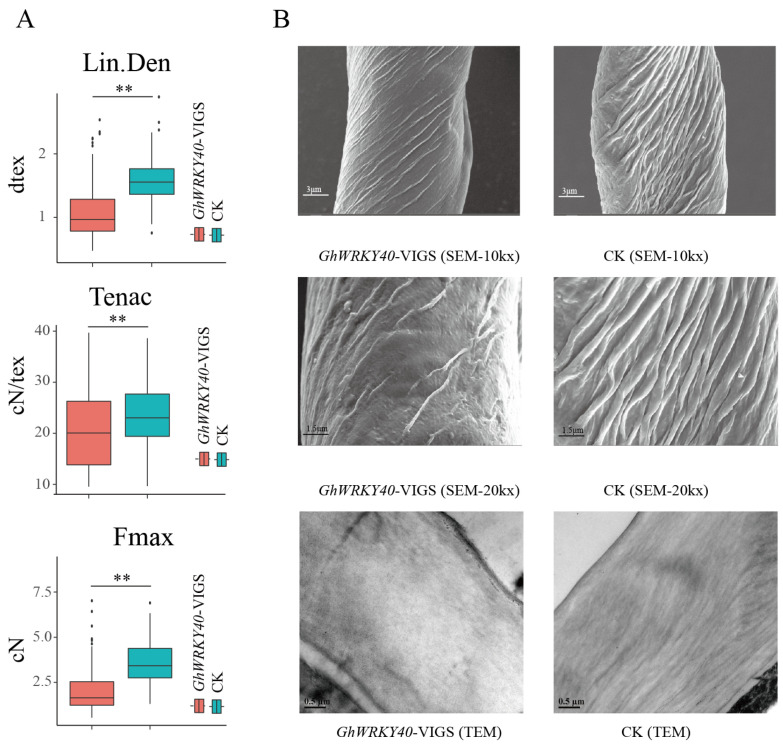
Variation in fiber quality indicators and changes in the fiber surface and internal structure in *GhWRKY40*-VIGS lines. (**A**,**B**): (**A**) Fineness of a single fiber (linear density, Lin.Den), strength of the fiber (Tenac), and force required to break a fiber (*F*_max_). One hundred fibers per replicate were measured, with three replicates analyzed. ** *p* < 0.01. (**B**): Electron micrographs of the changes in fiber surface and internal structure when *GhWRKY40* was silenced.

In our previous study, an increase in fiber length was observed when *GhWRKY40* expression was suppressed in the short fiber-length parent A29 [23]. Here, we used the VIGS system to validate the effect of *GhWRKY40* on fiber strength and fineness. To lessen the impact of external conditions and plant growth status on cotton fiber quality, we tested samples of blooming and flocculation from plants within three days of VIGS assay. The fiber strength indicators *F*_max_ and tenacity were significantly reduced in the *GhWRKY40*-downregulated lines compared with the control lines, and the Lin.Den data showed that the fiber fineness was significantly reduced to 65.35% of the control on average (Figure 1A). In the case of fiber determination using a high-volume instrument, the micronaire value is used to comprehensively measure fiber fineness and maturity [29]. In this study, we used Lin.Den to accurately measure the fiber thickness in a large number of replicates in a single fiber, and the effect of genes on fiber development was more precisely evaluated in this manner. The fibers were longer, finer, and weaker when *GhWRKY40* was downregulated; thus, we deduced that the significant thinning of the fibers may be one reason for the reduced fiber-breaking strength. In addition, the fiber surface was smoother, and the compactness of the internal structure of the fiber also changed in the *GhWRKY40*-downregulated lines (Figure 1B). These results suggest that GhWRKY40 may be involved in the regulatory pathway of fiber development, which affects fiber length [23], strength, and fineness.

### 3.2. Subcellular Localization of GhWRKY40

**Figure 2:**
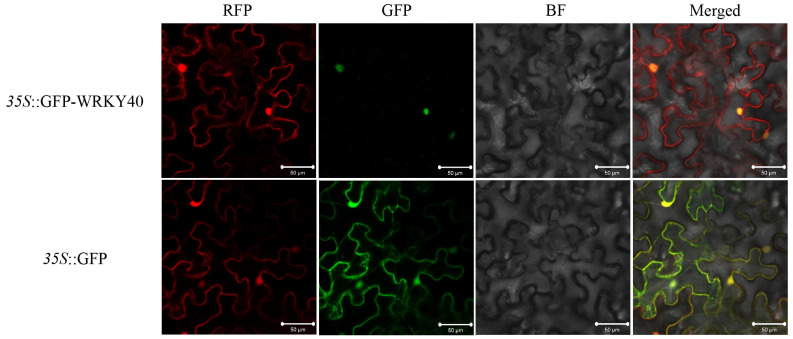
GhWRKY40 was functional in the nucleus. RFP: nucleoplasmic marker, GFP: green fluorescence field, BF: brightfield, and Merge: superimposed field.

To determine the functional location of *GhWRKY40*, transient expression vectors were created by fusing GFP with *GhWRKY40* under the control of the CaMV35S promoter and then delivered into *N. benthamiana* leaves. Fluorescence signals were observed to be localized only in the nucleus, indicating that GhWRKY40 was functional in the nucleus (Figure 2).

### 3.3. GhWRKY40-Interacting Proteins Were Screened in the Fiber Nuclear-System Y2H Library

**Figure 3:**
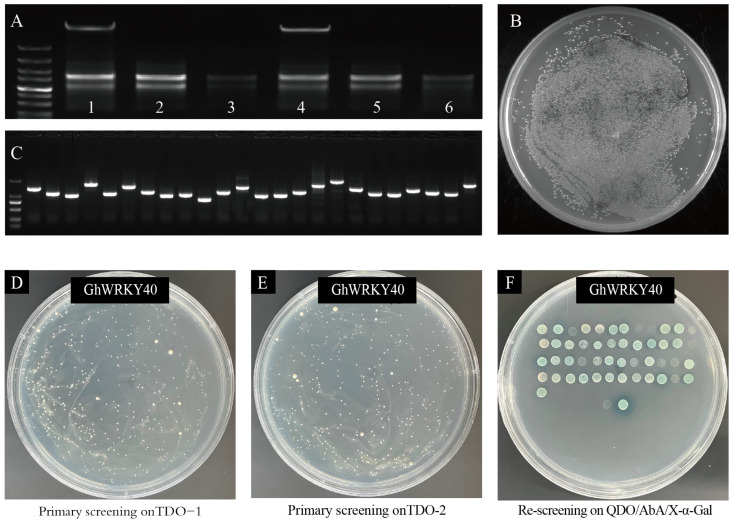
Construction of the cotton fiber nuclear-system Y2H library and the GhWRKY40-interacting protein screening. (**A**): Seven lanes from left to right are markers 1–6: A1-0 DPA, A1-10 DPA, A1-25 DPA, A29-0 DPA, A29-10 DPA, and A29-25 DPA. (**B**): Identification of the capacity of the secondary library of the nuclear system. (**C**): Identification of the size and recombination rate of the random insertion fragment of the nuclear-system library. (**D**–**F**): Primary screening and rescreening of GhWRKY40 interactors on the TDO and QDO/AbA/X-α-Gal media.

Total RNA was extracted from the cotton fibers of A1 and A29 at 0, 10, and 25 DPA and mixed in equal amounts, and then the total RNA was isolated, purified, and reverse transcribed to synthesize double-stranded cDNA for library construction. The primary and secondary cDNA libraries had 1.20 × 10^7^ CFU and 1.28 × 10^7^ CFU total clones, respectively, with a 100% positive combination rate, and the average insertion fragment of the yeast clone was more than 1000 bp (Figure 3A–C). The foregoing results suggested that the fiber nuclear-system Y2H library was well established and suitable for a two-hybrid screening.

To identify GhWRKY40-interacting proteins, pGBKT7-GhWRKY40 was used as the bait to screen the Y2H library of cotton fiber cDNAs constructed on the prey vector. The colony growth was consistent between pGBKT7-WRKY40 and the pGBKT7-empty vector on the SD/−Trp medium, demonstrating that the plasmid was not toxic to the yeast cells. Next, the positive control, the negative control, and the BD gene pGBKT7 WRKY40+pGADT7 were all cultured on DDO, TDO, and QDO media. The pGBKT7-WRKY40 gene showed slight self-activation activity in the Y2H Gold yeast strain and was used for the Y2H screening (Figure S1). In total, 49 clones were initially screened using the TDO medium, and then all were spotted onto a QDO/AbA/X-α-Gal medium, from which 42 positive clones were obtained after rescreening (Figure 3D–F). The cDNA sequences of the 42 clones were sequenced and blasted against the NCBI and CottonGen databases (https://www.cottongen.org/) (Table 2).

### 3.4. GhWRKY40 Fiber-Related Interactors Were Identified Using Y2H and Split-Luciferase Complementation Assays

The targets of GhWRKY40 proteins were associated with various aspects of plant development. Notably, some were putative fiber-related proteins, including the following: an R2R3-MYB protein (spot 10) and a fasciclin-like arabinogalactan protein (spot 18) involved in fiber initiation and elongation [13,30]; a BURP domain protein (spot 5) that may be associated with fiber quality in silico analysis [31]; a ubiquitin-conjugating enzyme (spot 22) that contributes to SCW thickening in *Arabidopsis* inflorescence stems, which is probably associated with fiber strength in cotton [32]; the cotton plasma membrane intrinsic protein 2 (PIP2, spot 25), which is associated with water transport and plays an important role in cotton fiber development [33]; an aspartyl protease/asparaginase, which may be associated with smooth fiber surfaces and convolutions (spot 45) [34]; and ATP synthase (spot 46), which is required to produce sufficient ATP for cotton fiber cell elongation [35] (Table 2).

**Figure 4:**
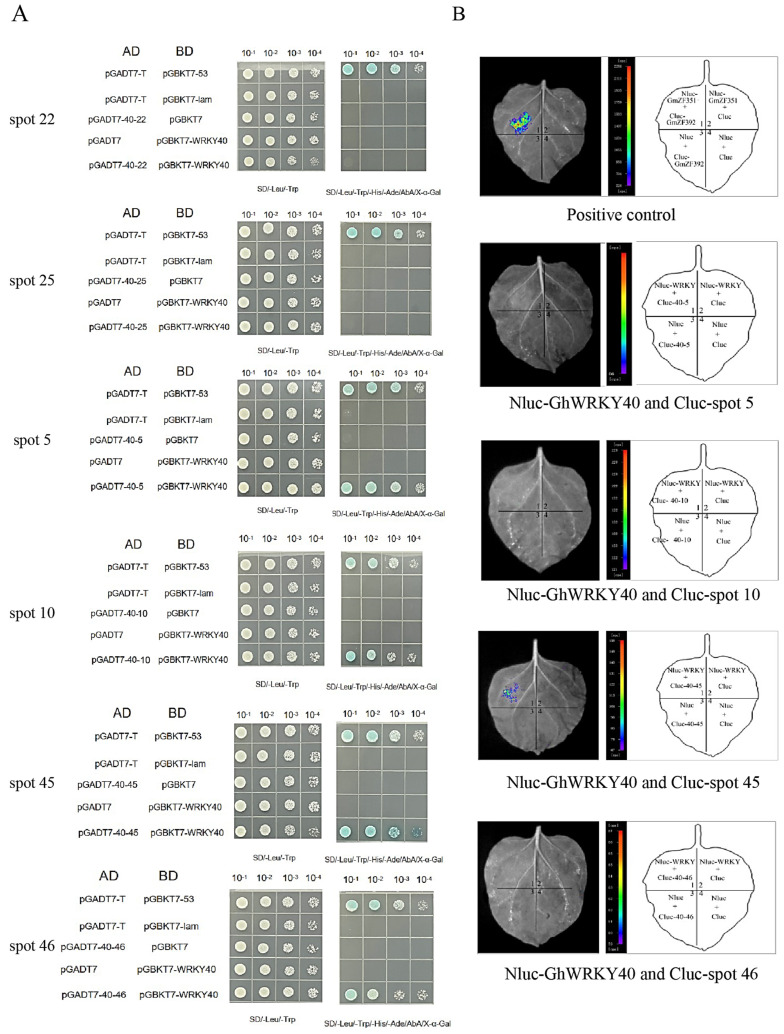
Screening of GhWRKY40 interactors in the Y2H and split-luciferase complementation assays. (**A**): Screening of GhWRKY40 interactors in the Y2H assays; BD was GhWRKY40 (abbreviated as WRKY40), and AD from top to bottom was located on spots 22, 25, 5, 10, 45, and 46. (**B**): Screening of GhWRKY40 interactors in the split-luciferase complementation assays.

All six probable fiber-related genes were chosen and amplified from A29, and the proteins were validated in one-to-one interaction analyses with the GhWRKY40 protein. In total, four of the proteins were observed to be GhWRKY40 interactors in the Y2H screens (Figure 4A). These four interactors were further evaluated in a split-luciferase complementation assay, but only an aspartyl protease/asparaginase *Ghi_D06G00926* (spot 45) could interact with GhWRKY40 (Figure 4B). The coding gene for the asparaginase is located on chromosome D6; therefore, it was designated *GhAP_D6_*.

### 3.5. GhWRKY40 May Regulate Fiber Development by Affecting the Activity of Aspartyl Protease

**Figure 5:**
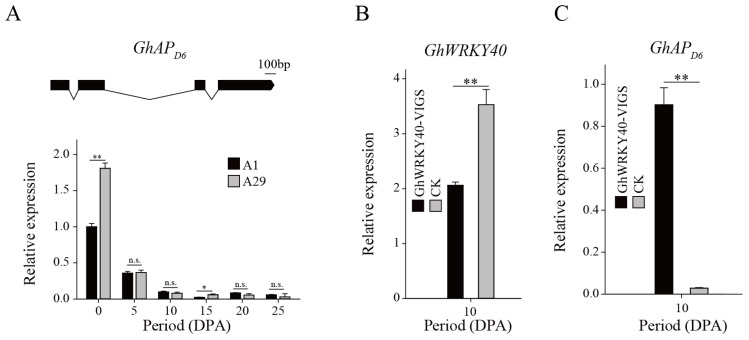
Gene structure and transcript level of *GhAP_D6_* in A1, A29, and *GhWRKY40*-VIGS lines. (**A**): Gene structure of *GhAP_D6_* and its transcript level in A1 and A29. (**B**): Transcript level of *GhWRKY40* was significantly downregulated at 10 DPA in A29. (**C**): Transcript level of *GhAP_D6_* was significantly upregulated when *GhWRKY40* was downregulated at 10 DPA in A29. * 0.01 < *p* ≤ 0.05, ** *p* ≤ 0.01.

**Figure 6:**
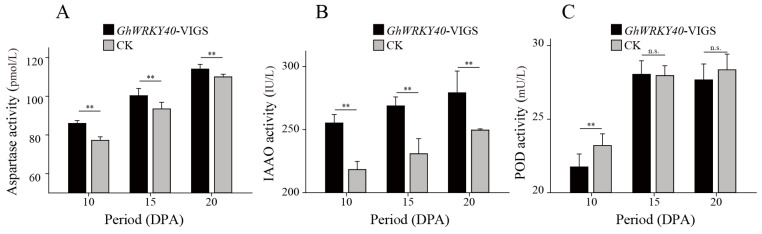
Aspartyl protease activity included the following: (**A**) indoleacetic acid oxidase (IAAO) activity; (**B**) peroxidase (POD) activity; and (**C**) activity when *GhWRKY40* was downregulated compared with that of the negative control. ** *p* ≤ 0.05.

The complete gene sequence of *GhAP_D6_* was amplified in both A1 and A29, but the *GhAP_D6_* sequence was identical in the two lines. The *GhAP_D6_* was 1984 bp in length and encoded 325 amino acids (Figure 5A). The *GhAPD6* transcript levels in fibers (ovules) from A1 and A29 were high at 0 DPA, significantly decreased from 0 to 10 DPA, and then remained low from 10 to 25 DPA (Figure 5A). When GhWRKY40 was significantly downregulated in A29 (Figure 5B), the aspartyl protease/asparaginase *GhAP_D6_* was significantly upregulated to approximately 30-fold higher levels in the *GhWRKY40*-VIGS lines at 10 DPA (Figure 5C). In addition, we measured the activity of the aspartyl protease in the *GhWRKY40*-VIGS lines between 10 and 20 DPA, which was correspondingly elevated after upregulation of the *GhAP_D6_* expression (Figure 6A). As a result, we deduced that GhWRKY40 may affect fiber development by regulating the activity of aspartyl protease/asparaginase.

Aspartic proteinases (APs; EC 3.4.23) have been implicated in various processes, such as secondary cell wall synthesis [36], stress responses [37], and fiber development [23,34]. Gul et al. [34] reported that overexpression of an asparaginase gene (*ZmASN*) in *G. hirsutum* resulted in a smoother fiber surface compared with that of a non-transgenic cotton line. In the present study, distinct upregulation of *GhAP_D6_* in the *GhWRKY40*-VIGS lines resulted in a similarly smoother fiber surface (Figure 1B). In *Populus tomentosa*, the aspartic protease PtoAED3 is involved in secondary xylem development, and transcriptome analysis in this study revealed that peroxidase expression is upregulated in *PtoAED3*-overexpressing materials, which is primarily reflected in changes in the total lignin content, which inhibits peroxidation [38]. In cotton, IAAO and POD play certain roles in regulating fiber development, such as cellulose synthesis, fiber elongation, and SCW thickening [39,40]. Considering the foregoing results, the IAAO and POD activities were measured in *GhWRKY40*-downregulated (*GhAP_D6_*-upregulated) lines; the IAAO activity was significantly upregulated between 10 and 20 DPA, whereas POD activity was slightly downregulated at 10 DPA (Figure 6B,C). In the present study, we found that GhWRKY40 interacts with an asparaginase GhAP_D6_ involved in fiber development in upland cotton. Our findings will assist in an understanding of the molecular mechanisms that govern cotton fiber growth as well as the molecular breeding of high-quality cotton fibers.

## 4. Conclusions

The transcription factor *GhWRKY40* affects cotton fiber strength, fineness, and surface smoothness. The asparaginase-related protein GhAP_D6_ may interact with GhWRKY40. The expression level of *GhAP_D6_* dramatically increased in the GhWRKY40-VIGS lines at 10 DPA, as did the aspartyl protease activity. This work suggests that GhWRKY40 may interact with aspartyl protease/asparaginase to regulate cotton fiber development. These molecular resources will be useful in molecular breeding to develop high-quality cotton fibers.

## Figures and Tables

**Table 1:** Fiber strength and fiber length of the *G. hirsutum* near-isogenic lines A1 and A29.

Number of Environments	Trait	A1		A29	
		Mean	SD	Mean	SD
5	Fiber strength (mm)	33.00	0.80	28.61	0.79
	Fiber length (mm)	31.49	0.69	28.88	0.73

**Table 2:** The cDNA sequences of 42 potential GhWRKY40-interacting positive clones screened in the fiber nuclear-system Y2H library.

No. of Spots	Protein Prediction	Blasted ID on NCBI
1	PREDICTED: GC2:C35 um glucose-6-phosphate isomerase 1, chloroplastic-like (LOC108489870), mRNA	Sequence ID: XM_017794574.2Length: 2420
2	PREDICTED: *G. hirsutum* RPM1-interacting protein 4 (LOC107961948), transcript variant X2, mRNA	Sequence ID: XM_041115985.1Length: 928
3	PREDICTED: *Gossypium arboreum* probable protein phosphatase 2C 33 (LOC108460622), transcript variant X2, mRNA	Sequence ID: XM_017760185.2Length: 2627
4	PREDICTED: *G. hirsutum* external alternative NAD(P)H-ubiquinone oxidoreductase B2, mitochondrial (LOC107900620), mRNA	Sequence ID: XM_016826287.2Length: 2190
5	PREDICTED: *G. hirsutum* BURP domain protein RD22 (LOC107958896), transcript variant X2, mRNA	Sequence ID: XM_041112951.1Length: 1357
6	PREDICTED: *G. hirsutum* pre-mRNA-splicing factor SPF27 homolog (LOC107902058), transcript variant X4, mRNA	Sequence ID: XM_016828321.2Length: 1136
7	PREDICTED: *G. hirsutum* pyruvate kinase, cytosolic isozyme (LOC107908997), mRNA	Sequence ID: XM_016836346.2Length: 1903
10	PREDICTED: *G. hirsutum* transcription repressor MYB6-like (LOC121210905), mRNA	Sequence ID:XM_041082841.1Length: 1236
11	PREDICTED: *G. hirsutum* 60S ribosomal protein L5 (LOC107954724), mRNA	Sequence ID: XM_016890373.2Length: 1293
13	PREDICTED: *G. hirsutum* nucleoside diphosphate kinase 2, chloroplastic (LOC107931375), mRNA	Sequence ID: XM_016863254.2Length: 959
14	PREDICTED: *G. hirsutum* elongation factor 1-α-like (LOC121230455), mRNA	Sequence ID: XM_041115288.1Length: 1680
15	PREDICTED: *G. arboreum* dnaJ protein homolog 1-like (LOC108459579), mRNA	Sequence ID: XM_017758969.2Length: 1702
16	PREDICTED: *G. hirsutum* 26S proteasome regulatory subunit 7A (LOC107906028), mRNA	Sequence ID: XM_016832870.2Length: 1554
17	PREDICTED: *G. hirsutum* uridine 5’-monophosphate synthase (LOC107903544), mRNA	Sequence ID: XM_016829617.2Length: 1816
18	*G. hirsutum* fasciclin-like arabinogalactan protein 12 (LOC107889256), mRNA	Sequence ID: NM_001326759.2Length: 1177
19	PREDICTED: *G. hirsutum* glyceraldehyde-3-phosphate dehydrogenase 2, cytosolic (LOC107899696), mRNA	Sequence ID: XM_016825467.2Length: 1560
20	PREDICTED: *G. hirsutum* cysteine synthase (LOC107893759), transcript variant X3, mRNA	Sequence ID: XM_041086207.1Length: 1350
21	PREDICTED: *G. hirsutum* bromodomain-containing protein 9-like (LOC107905723), transcript variant X2, mRNA	Sequence ID: XM_041094008.1Length: 2513
22	PREDICTED: *G. hirsutum* ubiquitin-conjugating enzyme E2 2 (LOC121209837), mRNA	Sequence ID: XM_041081548.1Length: 1129
23	*G. hirsutum* ATP synthase subunit delta’, mitochondrial-like (LOC107950798), mRNA; nuclear gene for mitochondrial product	Sequence ID: NM_001327581.2Length: 884
25	PREDICTED: *G. arboreum* probable aquaporin PIP2-8 (LOC108482897), mRNA	Sequence ID: XM_017785996.2Length: 1064
26	PREDICTED: *G. hirsutum* uncharacterized LOC107889703 (LOC107889703), transcript variant X2, mRNA	Sequence ID: XM_016814230.2Length: 1212
27	PREDICTED: *G. hirsutum* trafficking protein particle complex subunit 6B (LOC107961859), mRNA	Sequence ID: XM_016898021.2Length: 952
28	PREDICTED: *G. arboreum* SUMO-conjugating enzyme SCE1 (LOC108474073), mRNA	Sequence ID: XM_017775966.2Length: 932
29	PREDICTED: *G. arboreum* glyceraldehyde-3-phosphate dehydrogenase 2, cytosolic-like (LOC108456798), mRNA	Sequence ID: XM_017755477.2Length: 1619
30	PREDICTED: *G. hirsutum* elongation factor 1-α (LOC107922028), mRNA	Sequence ID: XM_016851852.2Length: 1697
31	PREDICTED: *G. hirsutum* uncharacterized LOC107899290 (LOC107899290), mRNA	Sequence ID: XM_016824958.2Length: 1353
32	PREDICTED: *G. hirsutum* uncharacterized LOC107925865 (LOC107925865), transcript variant X3, mRNA	Sequence ID: XM_016856595.2Length: 3458
33	PREDICTED: *G. hirsutum* bifunctional 3-dehydroquinate dehydratase/shikimate dehydrogenase, chloroplastic (LOC107888918), mRNA	Sequence ID: XM_016813187.2Length: 1970
36	PREDICTED: *Gossypium raimondii* proteasome subunit β type-1 (LOC105790336), mRNA	Sequence ID: XM_012617892.2Length: 1007
37	PREDICTED: *G. arboreum* V-type proton ATPase subunit E (LOC108450890), mRNA	Sequence ID: XM_017748687.2Length: 1291
38	PREDICTED: *G. hirsutum* eukaryotic translation initiation factor 3 subunit G (LOC107943593), transcript variant X1, mRNA	Sequence ID: XM_016877363.2Length: 1196
39	PREDICTED: *G. hirsutum* FKBP12-interacting protein of 37 kDa (LOC107926722), mRNA	Sequence ID: XM_016857634.2Length: 1477
40	PREDICTED: *G. hirsutum* nardilysin-like (LOC107954265), transcript variant X3, mRNA	Sequence ID: XM_041096636.1Length: 1232
41	PREDICTED: *G. hirsutum* protein EMBRYO DEFECTIVE 514 (LOC107912133), mRNA	Sequence ID: XM_016840199.2Length: 1086
42	PREDICTED: *G. hirsutum* S-adenosylmethionine synthase 1 (LOC107911137), mRNA	Sequence ID: XM_016839024.2Length: 1647
43	PREDICTED: *G. hirsutum* patellin-3 (LOC107904905), mRNA	Sequence ID: XM_016831418.2Length: 2448
44	PREDICTED: *G. hirsutum* lipoyl synthase, mitochondrial (LOC107920229), mRNA	Sequence ID: XM_016849822.2Length: 1613
45	PREDICTED: *G. hirsutum* probable isoaspartyl peptidase/L-asparaginase 2 (LOC107930584), mRNA	Sequence ID: XM_016862250.2Length: 1373
46	PREDICTED: *G. hirsutum* ATP synthase subunit O, mitochondrial (LOC107945533), mRNA	Sequence ID: XM_016879586.2Length: 1104
48	PREDICTED: *G. hirsutum* heat shock cognate protein 80-like (LOC107926182), mRNA	Sequence ID: XM_016856976.2Length: 2511
49	PREDICTED: *G. hirsutum* translocase of chloroplast 159, chloroplastic-like (LOC107933293), mRNA	Sequence ID: XM_016865463.2Length: 4119

## Data Availability

Data are contained within the article and Supplementary Materials.

## Supplementary Materials

The following supporting information can be downloaded at https://www.mdpi.com/article/10.3390/genes15080979/s1: Figure S1: toxicity and self-activation assay of pGBKT7-WRKY40; Table S1: primers used in this study.
