# Supplementary material for: GhWRKY40 Interacts with an Asparaginase GhAPD6 Involved in Fiber Development in Upland Cotton (Gossypium hirsutum L.)
Source: Genes (Basel). 2024 Jul 24;15(8):979. doi: 10.3390/genes15080979 (PMC11353873; doi:10.3390/genes15080979)
Supplement: Supplementary file 1 [file genes-15-00979-s001.zip › Figure S1.pdf]

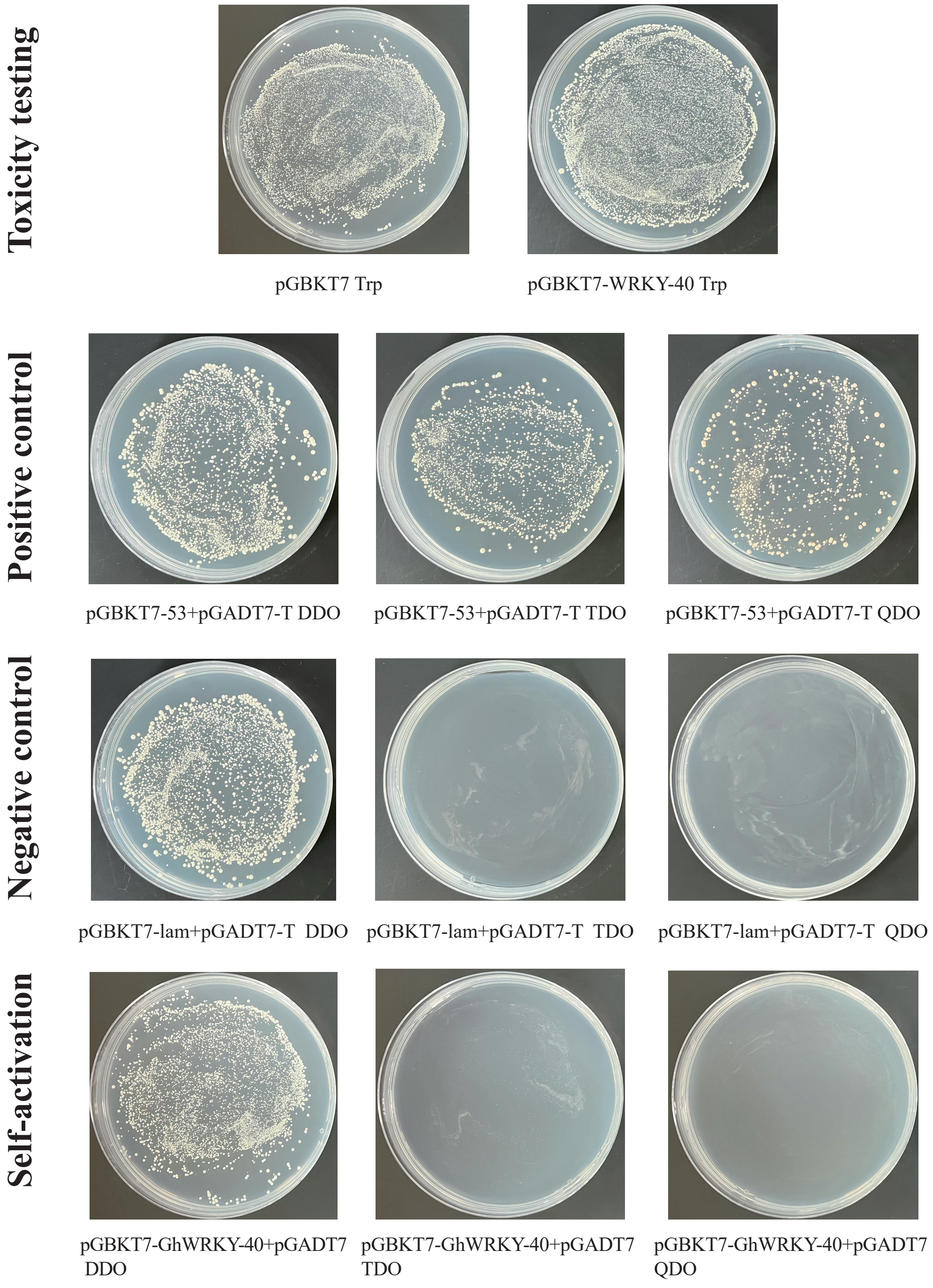

Toxicity testing
pGBKT7 Trp
pGBKT7-WRKY-40 Trp
Positive control
pGBKT7-53+pGADT7-T DDO
pGBKT7-53+pGADT7-T TDO
pGBKT7-53+pGADT7-T QDO
Negative control
pGBKT7-lam+pGADT7-T DDO
pGBKT7-lam+pGADT7-T TDO
pGBKT7-lam+pGADT7-T QDO
Self-activation
pGBKT7-GhWRKY-40+pGADT7 DDO
pGBKT7-GhWRKY-40+pGADT7 TDO
pGBKT7-GhWRKY-40+pGADT7 QDO
